# Study Protocol on Hormonal Mediation of Exercise on Cognition, Stress and Immunity (PRO-HMECSI): Effects of Different Exercise Programmes in Institutionalized Elders

**DOI:** 10.3389/fpubh.2016.00133

**Published:** 2016-06-27

**Authors:** Ana Maria Teixeira, José Pedro Ferreira, Eef Hogervorst, Margarida Ferreira Braga, Stephan Bandelow, Luís Rama, António Figueiredo, Maria João Campos, Guilherme Eustáquio Furtado, Matheus Uba Chupel, Filipa Martins Pedrosa

**Affiliations:** ^1^Faculty of Sport Sciences and Physical Education, University of Coimbra, Coimbra, Portugal; ^2^National Centre for Sports and Exercise Medicine, Loughborough University, Loughborough, UK; ^3^Medical Psychology Unit, Department of Clinical Neurosciences and Mental Health, Faculty of Medicine, Porto University, Porto, Portugal; ^4^CAPES Foundation, Ministry of Education, Brasilia, Brazil

**Keywords:** exercise, older women, cognition, immunity, mental health

## Abstract

Physical activity (PA) in elders has been shown to have positive effects on a plethora of chronic diseases and to improve immunity, mental health, and cognition. Chronic stress has also been shown to have immuno-suppressive effects and to accelerate immunosenescence. Exercise could be a significant factor in ameliorating the deleterious effects of chronic stress, but variables such as the type, intensity, and frequency of exercise that should be performed in order to effectively reduce the stress burden need to be defined clearly. PRO-HMECSI will allow us to investigate which hormonal and immunological parameters are able to mediate the effects of exercise on mucosal immunity, psychological/biological stress, and cognitive functioning in older people. Phase I consists of an observational cross-sectional study that compares elders groups (*n* = 223, >65 years) by functional fitness levels aiming to identify biomarkers involved in maintaining immune and mental health. Neuroendocrine and immune biomarkers of stress, psychological well-being related to mental health, neurocognitive function, functional fitness, and daily PA will be evaluated. Phase II consists of a 28-week intervention in elders with mild cognitive impairment (MCI) profile (*n* = 149, >65 years, divided in three groups of exercise and one control group), aiming to investigate whether the positive effect of three different types of chair-based exercise programs on physical and psychological health is mediated by an optimal endocrine environment. Primary outcomes are measures of cognitive function and global health. Secondary outcomes include the evaluation the other dimensions such as immune function, psychological health, and depression. Few studies addressed the effects of different types of exercise interventions in older population samples with MCI. We will also be able to determine which type of exercise is more effective in the immune and hormonal function of this population.

## Introduction

Mild cognitive impairment (MCI) refers to a stage in which a person experiences memory loss to a greater extent than one would expect for that age, but do not yet meets currently accepted criteria for clinically probable Alzheimer disease (AD) or other types of dementia (1, 2). The recently coined concept of frailty – decreased resistance to stressors and increased risk for adverse health outcomes (3), has been reported to modulate the risk of several types of dementias and cognitive impairment *tour court*. Cognitive and physical frailty may be divided in subtypes according to their reversibility and task forces are proposing refined criteria to detect frail older subjects (4). Vascular, inflammatory, nutritional, and metabolic factors appear to be involved in late life frailty and are targeted as preventive causes of dementia (5). Recently published longitudinal studies report that muscle mass and physical performance may constitute an independent frailty component, according to the type of dementia, pointing to the need of further research (6). In general, the relationship between general cognitive function and physical frailty has been well documented (7). Although less consistently with dementia (8), but some authors argue that a dynamic perspective conveyed by longitudinal approaches of frailty may contribute to better understand transitions in cognitive status (9).

Immunosenescence is also a part of the aging process and is associated with an increased risk for autoimmune disorders, tumors, or infectious disease and neurocognitive disorders (10). The mucosal immune system, including the upper respiratory tract, is considered a first barrier to the colonization by pathogenic agents, reducing the incidence of upper respiratory tract infections (URTI) in humans (11). During aging, there is a decrease on salivary immunoglobulin-A (sIgA) secretion which is also linked to higher URTI incidence (12). Engaging in regular sports, exercise or systematic physical activity (PA) is known to protect against many factors associated with poor physical and psychological health and improves life expectancy (13). Cognitive functions sensitive to early dementia and age-related cognitive decline, such as memory and executive functions and simple and complex information processing reaction times have been shown to respond and be sensitive to the effects of exercise in both young and old ages (14). Six months of moderate levels of aerobic activity were sufficient to produce significant improvements in cognitive function, with the most dramatic effects occurring on measures of episodic memory and executive control (15). Dementia and vascular disease are leading causes of mortality in women, but risk seems to be reduced with exercise (16). Depressive symptoms are present in 12–30% elders (17), frequently associated with cognitive impairment (18). Furthermore, the presence of immune system dysfunction and inflammation has been reported to predict depressive symptoms during aging and appears to be the link between depression and dementia (19). It is very important for public health to identify the main factors associated with cognitive decline in the elders, subsidizing the creation of new methods of therapy. Exercise may have the largest positive effects on older women who have low levels of sex steroids after menopause which at the same time increase the risk for cardiovascular disease and dementia (20). The type of exercise seems to be crucial on increasing the aerobic capacity, but the evidence to support the involvement of flexibility, aerobic, and strength resistance exercise in improving cognition and psychological well-being related to mental health are not clear (14), and their effectiveness should to be investigated. Promising results have been reported in the reduction of depressive symptoms with PA interventions when including flexibility/resistance and low intensity exercise (21). In the older persons, PA appears to be beneficial for those presenting clinical depression, although the effect of different programs is still unclear (22). A few studies have looked at the effect of regular moderate exercise on SIgA, and there is no sufficient evidence to support the effect of exercise on IFN-γ and C-reactive protein (CRP) levels in elders (10). Regular exercise has also been shown to diminish the level of stress and anxiety and the risk of psychological diseases and emotional decline in elders (23). The responses of saliva flow rate and their composition during exercise are influenced by sympathetic nervous system activity and the hypothalamic–pituitary–adrenal axis (HPA-axis), the salivary glands being enervated by both parasympathetic and sympathetic nerves (24). Recent studies have identified salivary α-amylase (α-Amy) as a potential marker of sympathetic activity, while salivary cortisol (sCor) seems to be a valid measure for the HPA-axis activity (25). α-Amy is an enzyme that catalyzes starch into maltose and can be important to host defense by inhibiting the adherence and growth of certain bacteria (26). Testosterone levels decline with aging as well as cognitive function, and their levels seems to be diminished in patients with AD and MCI (27). Dehydroepiandrosterone (DHEA) is another steroid hormone involved in metabolism, produced mainly in the adrenal cortex, its functions are linked with anti-glucocorticoid, anti-oxidant anti-inflammatory, and immunomodulatory effects (28). Recently, DHEA has been investigated for its relationship to mental and physical stress and also in psychological and behavioral disorders. The DHEA plasma concentration and the ratio SCor/DHEA have also been shown to increase with PA (29). Salivary markers may serve as potential non-invasive tools for evaluation of the relationship between the central nervous system and mucosal immunity following psychological and/or physical stress and how these may affect cognitive functions (24).

In addition to these measures in saliva, peripheral blood concentrations of inflammatory biomarkers, such as interleukin-1 beta (IL-1β), tumor necrosis factor alpha (TNF-α), and interleukin-6 (IL-6), have previously been found to be elevated in cases of MCI and depression in comparison to healthy age-matched controls (23, 24, 29). Evidence shows that immunological and hormonal parameters are able to mediate the effects of exercise on mucosal immunity, psychological stress, cognitive improvement, and risk of dementia in the elders who are regularly active (30) and that regular exercise may provide an effective strategy in the treatment and prevention of associated disorders by anti-inflammatory benefits. IL-1β, IL-6, IL-10, IFN-γ, TNF-α, and CRP are important immune markers that interact in anti- and proinflammatory processes triggered by aging, and affecting the cognitive profile of the elderly. Studies have implicated the inflammatory pathway on increased severity of white matter hyperintensities and brain atrophy as mechanisms to develop brain alterations and cognitive decline in older subjects (31, 32).

Highlighted as an important neuroendocrine marker, the brain-derived neurotrophic factor (BDNF), is involved in neuroplasticity, differentiation, neuronal growth, learning, and memory (33, 34). Its decrease is reported in individuals with Parkinson’s, AD, depression, and MCI, this last one a clinical condition evidenced through cognitive testing (35). Differences in BDNF polymorphisms may have consequences on antidepressant efficacy (36), and the presence of depressive symptoms is associated with lower BDNF peripheral levels before antidepressant treatment (37). Exercise can act as a positive mediator of cognitive functioning in individuals suffering from early dementia and mental disorders, these responses being attributed to a possible role of BDNF (15, 38).

## Aims of the Study

(a) To examine the multivariate associations between physical fitness (PF), cognitive indexes, biomarkers of inflammation, stress, and neurotrophic factors, associated with psychological well-being and mental health, in a cross-sectional study in older women subjects;
(b) To verify the hypothetical effect of different types of regular exercise practice on PF, hormonal responses related to immunological and neurocognitive systems in healthy older participants and those with MCI after 14 and 28 weeks of regular practice;
(c) To explore the associations between changes in neurocognitive and immunological systems, functional fitness, psychological well-being, and mental health by different exercise programs after 14 and 28 weeks of regular practice.

## Initial Procedures

**Figure 1:**
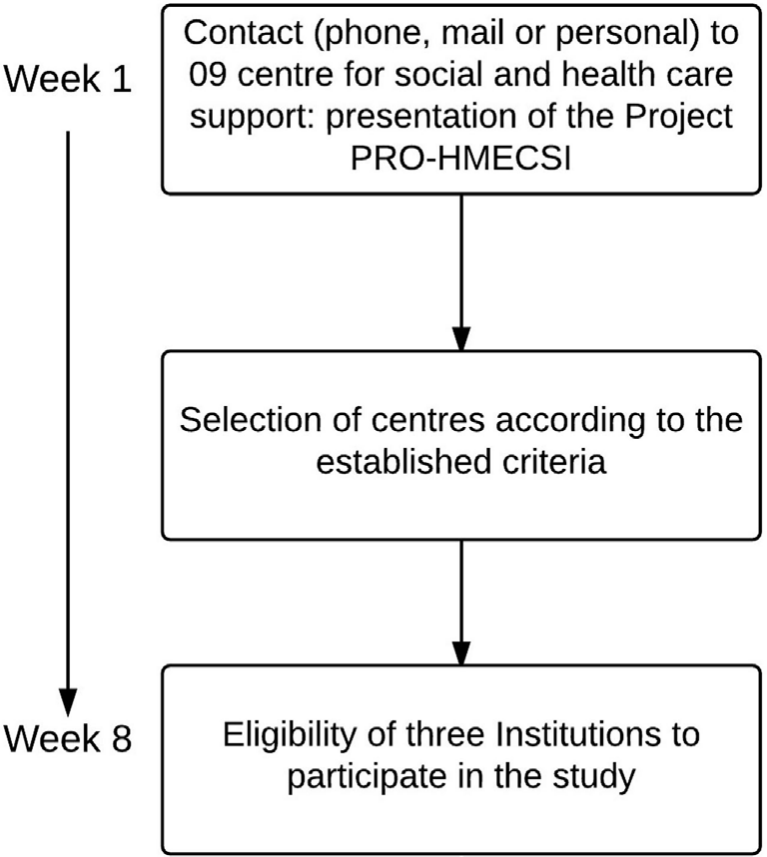
**Flow chart of the PRO-MHESCI study design**.

All CSHS and participants (or responsible) will be required to give a full informed consent before beginning the study. Individuals who express interest in participating in the program will sign a statement of responsibility, in which the privacy of identity and data collected will be guaranteed as well as the possibility of accessing the medical report of the subject. The contact with the medical center was established as one of the criteria to verify the eligibility of each subject to perform the exercise program (Figure 1). Once individuals were interviewed to participate in the study, they were informed of the procedures for data collection and of the class sessions that would occur in the pilot study. An information session about the study protocol was created for presentation to local organizations and to all the individuals that express interest in participating in the study. The study protocol has been approved by Faculty of Sport Sciences and Physical Education Ethical Committee – University of Coimbra (number reference: CE/FCDEF-UC/000202013), and it is integrated in the research project entitled “PRO-HMECSI: Hormonal mediation of exercise on cognition, stress and immunity”; respected the Portuguese Resolution (Art.° 4st; Law no. 12/2005, 1st series) on ethics in research with humans (39); follows the guidelines for ethics in scientific experiments in exercise science research (40) and still, complied with the guidelines for research with human beings of the Helsinki Declaration (41).

### CSHS Eligibility Criteria

CSHS eligibility criteria include (a) ability to participate in a study with total duration of 12 months and encompassing three phases: cross-sectional study, intervention, and detraining periods; (b) existence of an appropriate physical space to carry out the exercise sessions; and (c) required support of caregivers to assist with the elders displacement to the exercise classes.

### Older Participant’s Eligibility Criteria

The inclusion conditions for the older participants stipulated in order are being a female participant aged over 65 years; drug therapy controlled and updated; if the participant presents a clinical condition or comorbidity, it must be stable and enable participation in the exercise classes as decided by local medical staff. Specific criteria for participant exclusion are not completing or withdrawing from the “8-foot-up-and-go test” (8-UGT) in the maximum time of 50 s. According to previous studies with samples of institutionalized elderly, scores above this value indicate severe disability/mobility dependence (42); involvement in other structured exercise programs; the presence of any type of health condition that could prevent testing of functional autonomy, such as severe cardiopathy, hypertension, uncontrolled asthmatic bronchitis, and any musculoskeletal conditions that might prevent testing (i.e., osteoarthritis, recent fractures), mental disorder, hearing and vision impairment, morbid obesity, or the use of medications that could cause high attention impairment.

## Methods/Design

This research is planned for ~16 months, and it is built in three different phases/studies as described below: the cross-sectional study 1 (4 weeks’ duration) consists in the evaluation of older people (≥65 years old) aiming to investigate existing multivariate associations between PF, psychological well-being, cognitive, immunological, and neurotrophic factors in institutionalized elderly woman. Participants are older women living in CSHS. We will contact these subjects in CSHS located in the city of Coimbra, Portugal. Primary outcomes will be collected by a short test battery that measures biosocial, global health, cognitive, and PF indicators. PF battery, psychological well-being, multidimensional cognitive function profile, immunological, and neurotrophic factors are second outcomes and will also be collected. In addition, the secondary goals of this cross-sectional study 1 will be to identify the older women with MCI, for subsequent stratification and participation in the intervention study 2, described below.

**Figure 2:**
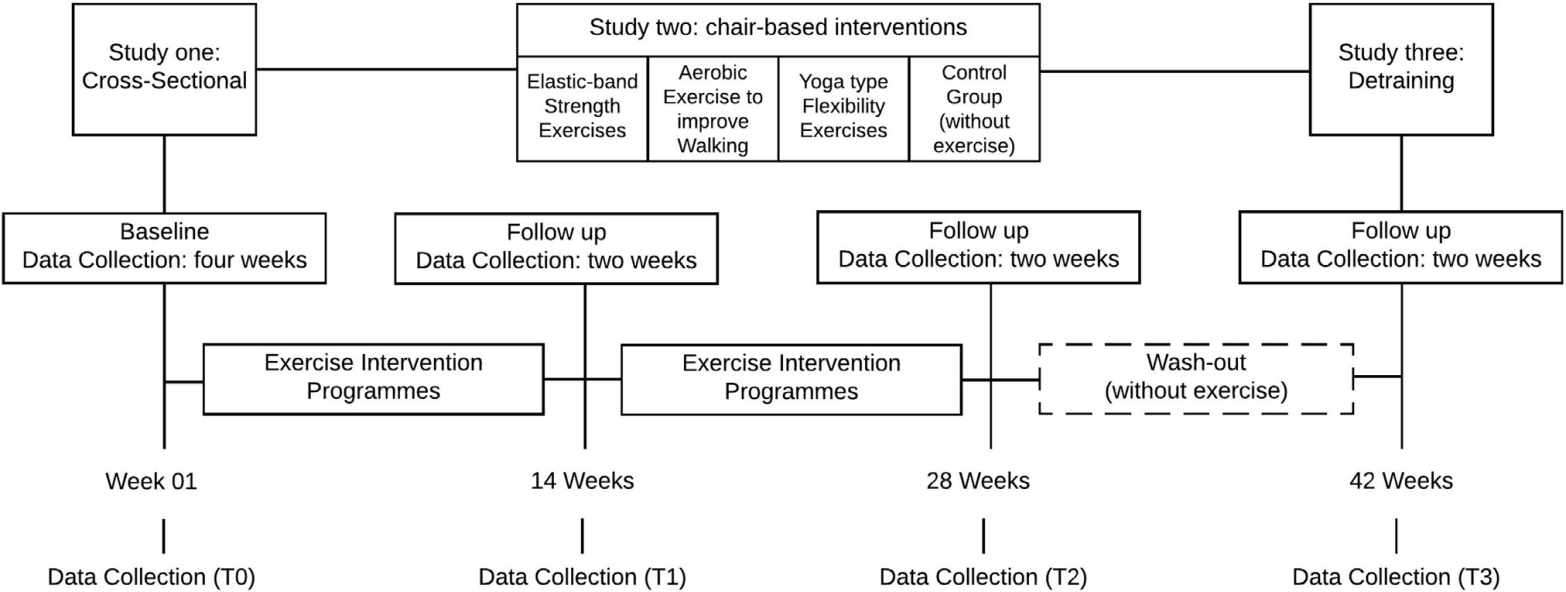
**Timeline of the PRO-MHESCI: study 1 (cross-sectional), study 2 (Intervention), and study 3 (washout period)**.

The observational study 2 is an intervention study with three different chair-based exercise (CBE) programs for women aged ≥65 years. The study is designed to assess the effect of strength/elastic band, aerobic/walking, and yoga/flexibility exercise interventions on secondary outcomes of PF, immunological, and neurotrophic markers as well as cognitive function in older women with MCI, recruited in institutionalized context. It is assumed that the effect of the exercise programs is independent. Measurement of primary outcomes will take place 4 weeks prior to the beginning of the exercise programs, on the same variables of study 1. Participants in these groups will attend a 45-min exercise session, 2–3 times/week during 28 weeks. The exercise programs will be run in the care centers for safety, disability, and comfort reasons. An appropriate space will be prepared by our fitness instructor’s team in each center to run the exercise sessions. Study 3 will involve analyzing the effect of 14 weeks detraining in all variables described in the observational study 2 above. Data regarding the assessment of psychological well-being related to mental health, cognitive function, PF, and biochemical markers will be collected (Figure 2).

## Sample Size

Sample size was estimated considering data of sample sizes described in a recent review (43) that obtained significant results involving the effects of exercise on immunological and cognitive parameters of elderly samples with MCI. Estimating the incidence of MCI in institutionalized elders to be around 30 and 57% (44), a total of 223 participants will be recruited for study 1. The sample size for study 2 was estimated adjusting MANOVA for repeated measures effects, alpha (Type I error rate) at 0.05, and power (Type II error rate) at 0.85 were computed using G* Power Version 3.1.9.2 (45, 46). A total of 149 participants are estimated to provide enough information about the outcome variability in our study design. It is predicted that only 70% of the participants will complete the full program (47) justifying a minimum sample size of 25 individuals for both intervention and control group.

## Exercise Adherence

Exercise sessions will be offered 2–3 times/week, during 28 weeks, in a total of 78 sessions. The percentage of exercise adherence to group classes is calculated individually through the total sum of participation. Entries will be recorded in a database. When a participant has two consecutive absences, she will be contacted to return to the group classes. According to a recent systematic review, an adherence to the exercise program of 55% was established as minimum for each participant to be included in the study (47).

## Pilot Study

A pilot study was conducted during 4 weeks with an exercise session per week. At the same time, interviews, psychometric scales, cognitive, and functional tests were applied to check the subject’s conditions and evaluate the methods of application of the study. Classes with duration of 30 min were given during this period (pre-training, easy-level), to gain insight to the implementation of CBE programs, adequacy of the spaces and structures where the classes would take place, and test the rating of perceived exertion by the participants.

## Masking

The fitness instructor of the all CBE sessions will not take part in data collection procedures. Collection of saliva and blood samples as well as the global health assessment will be performed by a registered nurse. The assessment of psychometric, cognitive scales, and physical–functional fitness battery was organized by the principal investigators and will be applied by specialists and co-investigators of the research team. This assessment can be done in different days, respecting the motivation of the elders, interrupting the test, and continuing on another day if they feel tired or uncomfortable. To minimize differences in procedures the same evaluators that will perform the data collection will apply both baseline and follow-up questionnaires and physical–functional fitness battery tests. The specialists of PF, psychometric, and cognitive assessments will not make any reference to the exercise program and do not have access to the remaining data.

## Outcome Measures

All outcome measures will be collected at baseline (study 1). After 14 and 28 weeks of exercise intervention (study 2) and after week 42 (washout period), biosocial and global health status will not be included. The primary outcomes are measures of cognitive function, biosocial, and global health status. One session will be used to administer a short test battery to measure biosocial, global health status, cognition (MCI screening), and PF (mobility screening) in this specific order. Secondary measures comprise psychological well-being, cognition, PF, anthropometric, immunological, and neurotrophic markers.

## Biosocial Status

Information on sociodemographic characteristics such as chronological age (continuous and category form), gender (category), and education (continuous and category) will be collected and used for adjusting models.

## Neurocognitive Profile I: Memory Complaints and MCI Screen

Participants included will be institutionalized older adults with memory complaints, i.e., those with possible MCI, who are at risk for developing dementia. For this purpose, the combination of two cognitive tests and respective cut-off values will be used.

## Mini Mental State Examination

The mini mental state examination (MMSE) assesses five areas of cognition: orientation, immediate recall, attention and calculation, delayed recall, and language (48). The maximum score is 30 points and a score below 24 points is considered abnormal and used for dementia and MCI screening (49). The MMSE will be used to classify participants by cognitive profile as a category variable, following the criteria described by Mungas (1): (a) severe cognitive impairment (SCI, values between 01 and 09), (b) moderate cognitive impairment (MOCI, between 10 and 18); (c) MCI (values between 19 and 24); (d) normal cognitive profile (NCP, values between 25 and 30). The MSSE was included because it was shown to be sensitive to the effects of exercise in an older population (50).

## Hopkins Verbal Fluency Test

The Hopkins verbal learning task (HVLT) is one of the most commonly used memory tests in clinical neuropsychological evaluation of older adults and is used to assess verbal episodic memory, including immediate memory (48). It is a 4-min test, easy to administer, to score, and to well tolerate even by significantly impaired individuals. This test requires recall of a series of 12 words (nouns) from 3 semantic categories (precious stones, animals, and human dwellings) over 3 learning trials (35). Scores between 15.5 and 24.5 on this test indicate a risk of dementia or MCI (48). Recent studies indicate that this test has satisfactory construct and concurrent validity and good test–retest reliability, range 0.80 to 0.98 values (51, 52).

## Global Health Status Evaluation

This evaluation includes the initial contact with the medical staff of the CSHS, in order to collect information on the medical history and current health of the participants that may condition their participation in the exercise programs. Additionally, comorbidity severity will be evaluated using the Charlson comorbidity index (CCI), in association with the medical drug record of the elders.

## Charlson Comorbidity Index

The CCI is a method of predicting mortality by classifying or weighting comorbid conditions that has been widely utilized by health researchers to measure burden of disease. It has a weighted index based on 17 comorbid conditions that has been shown to predict 1- and 10-year mortality (53, 54). A recent studied aimed to update the index of 12 comorbidities showed adequate discrimination in predicting and classifying comorbidities, when analyzing data from six countries (55). In this study, the total score of the ICC is used as a continuous variable.

## Functional Fitness Assessment I: Mobility Screen

To assess quickness, agility, and dynamic balance, the “*8-foot up-and-go test”* (8-UGT) will be used. The time needed for the participant to get up from the chair, walk as quickly as possible around either side of the cone, and to sit back down in the chair is registered (56). According to recent research, the 8-UGT can be used to effectively screen older people at risk for low mobility (57, 58).

## Secondary Outcomes

In total, three sessions will be used for collection of secondary outcomes. In the first session, cognitive and psychological tests will be applied. In the second day, blood and saliva samples will be collected. Anthropometric measures and PF tests will be applied in the third session.

## Neurocognitive Profile II: Multidimensional Cognitive Screen

The cognitive profile assessment will be evaluated using the Portuguese version of the multidimensional evaluation cognitive battery developed by Hogervorst–Bandelow and used worldwide in many treatment and observational studies (13, 32). The cognitive testing requires <20 min, including MMSE and HVLT. These tests have in earlier interventions also shown to respond and be sensitive to the effects of exercise in the elders (59).

## Verbal Fluency Test

Verbal fluency is a cognitive function that enables the retrieval of information from memory linked to executive and linguistic abilities (60). The verbal fluency test (VFT) evaluates an individual’s ability to retrieve specific information within restricted search parameters, such as the semantic fluency, tested by asking the participant to generate a semantic category with names of animals (61). This test consists of giving the person 60 s to verbally list as many animals as possible.

## Digital Span Test

The digital span test (DST) consists of two tests: in the first one, the examiner says a series of numbers and asks the participant to repeat them back in the same order. The test finishes when the subject fails two times in the same series or completes all the series (up to nine numbers). In the second one, the subject is asked to repeat a series of numbers in the inverse order. The first series begins with two numbers, and then continues in the same manner by increasing one number at each time. The test finishes when the subject fails two times in the same series or completes all the series (up to eight numbers). Administering the test forward assesses both attention and short-term memory. When the backwards version of the test is given, it also measures working memory (62).

## Symbol Modality Test

This is a measure of attention, perceptual speed, motor speed, visual scanning, and memory. A piece of paper with nine symbols corresponding to nine digits is given to the participants. On another sheet of paper, there are several rows of digits with empty spaces below them. The subjects are asked to fill in as many corresponding symbols as possible in 90 s (63).

## Psychological Well-Being/Mental Health

The test battery will be administrated by the same research team after a short briefing on the purpose of the study and requires <10 min. Standardized instructions will be given to all participants as well as encouragement to ask for help. Individual attention will be provided to participants with interpretation doubts, questions will be read to clarify the meaning assuring that no emphasis will be put on the question in order to avoid directing the answer. The test battery to be used in this study includes the Portuguese version of the tests describing below. All the tests scores will be analyzed as continuous variables.

## Rosenberg Self-Esteem Scale

A 10-item scale that measures global self-worth by measuring both positive and negative feelings about one self. The scale is believed to be unidimensional. All items are answered using a four-point Likert scale format ranging from “strongly agree” to “strongly disagree” (64). For items 1, 2, 4, 6, and 7, a reversal of the scores is done. The global self-esteem is represented by the sum of all scores and gives results between 10 and 40 points, where higher values represent higher levels of global self-esteem. Many studies have shown it to respond and be sensitive to the effects of exercise in the elders (65).

## Satisfaction with Life Scale

The satisfaction with life scale (SWLS) is a short five-item instrument designed to measure global cognitive judgments of satisfaction with one’s life. The scale usually requires only about 2 min of the participants’ time (66). It uses a seven-point Likert scale, indicating your agreement with each item by placing the appropriate number on the line preceding that item. Results range between 1 and 35 points, with higher values representing higher levels of life’s satisfaction (67).

## Perceived Stress Scale

The perceived stress scale (PSS) was originally developed as a 14-item scale that assesses the perception of stressful experiences by asking the participant to rate the frequency of his/her feelings and thoughts related to events and situations that occurred over the previous month. Seven out of the 14 items of PSS-14 are considered negative and the remaining 7 as positive, representing perceived helplessness and self-efficacy, respectively (68). For items 4, 5, 6, 7, 9, 10, and 13, a reversal of the scores is done. Final scores vary from 14 to 70 points. A higher score indicates greater stress (69).

## Hospital Anxiety and Depression Scale

This questionnaire consists of two subscales, one measuring anxiety, with seven items, and one measuring depression, with seven items, which are scored separately. Each item is answered on a 4-point (0–3) response category, so that the possible scores range from 0 to 21 for anxiety and from 0 to 21 for depression. It takes 2–5 min to complete. The hospital anxiety and depression scale (HADS) manual indicates that a score between 0 and 7 is “normal,” between 8 and 10 “mild,” between 11 and 14 “moderate,” and between 15 and 21 “severe” (70).

## The Lawton Instrumental Activities of Daily Living Scale

The Lawton scale is a suitable questionnaire to assess independent living skills. The instrument is most useful for identifying how a person is functioning at the present time and for identifying improvement or deterioration over time (71). There are eight domains of function measured with the Lawton independence activities day living (IADL) scale. Current recommendations are to assess all domains for both genders, although cultural differences with regard to the proposed tasks to be evaluated may exist. Participants are scored according to their highest level of functioning in that category (72). A summary score ranges from 0 (low function, dependent) to 8 (high function, independent).

## Katz Index of Independence in Activities of Daily Living

The Katz index of independence in activities of daily living (ADL) is the most appropriate instrument to assess functional status as a measurement of the older person’s ability to perform ADL independently (73). The index ranks adequacy of performance in the six functions of bathing, dressing, toileting, transferring, continence, and feeding. Participants are scored yes/no for independence in each of the six functions (74). A score of 6 indicates full function, 4 indicates moderate impairment, and 2 or less indicates severe functional impairment (75).

## Falls Efficacy Scale

The falls efficacy scale (FES) contains questions that assess the concern about the possibility of falling during the performance of 10 activities (76). The trust that the elders have to perform the activities without falling is represented on a 10 points analog scale ranging from “No confidence” (10 points) to “Completely confident” (1 score). The score of the FES is the sum of the scores obtained in each of the 10 items. The minimum score possible is 10 and the maximum is 100. Accordingly, the lower the score, the greater the confidence, resulting in a high self-efficacy (77).

## Biochemical Assessment I: Collection of Saliva Sample

Saliva will be collected by passive drool (the participant allows saliva to collect on the floor of the mouth, then leans forward and dribbles into a tube), for 3 min in high quality polypropylene vials to avoid problems with analyte retention or the introduction of contaminants that can interfere with the immunoassays. The collection times are always at the same time in the morning in order to minimize the circadian effect seen with some of the markers under study. Prior to the saliva collection subjects will be asked to rinse their mouth with water to remove food residues 10 min before sample collection and to avoid: alcohol for 12 h, dairy products for 20 min, a big meal for 60 min, foods with high sugar or acidity, or high caffeine content immediately before sample collection. The tubes containing the saliva will then be frozen, then defrosted and centrifuged in order to collect the saliva sample. The volumes measured, the flow rate calculated, and the samples will be store at −20°C until determination of the saliva markers proposed for this study. IgA, testosterone, cortisol, and DHEA will be analyzed by ELISA (Salimetrics, UK), and Alpha-Amylase by a kinetic-assay (Salimetrics, UK), according to standard procedures (78).

## Biochemical Assessment II: Collection of Blood Sample

Blood will be collected by venopuncture, in a fasted state, by a registered nurse. Determination of blood counts done after the blood collection, and then the tubes will be centrifuged for the collection of plasma and serum and these stored in cryovials at −80°C until determination of the serum and plasma markers proposed for this study. The levels of the pro- and anti-inflammatory cytokines, such as IL-1β, IL-6, IL-10, IFN-γ, and TNF-α, the cardiovascular risk marker, such as CRP, and the neurotrophic factor, such as BDNF, will be analyzed by ELISA kits according to the manufacturers’ instructions.

## Functional Fitness Assessment II: Senior Functional Battery

The functional fitness of every participant will be measured using the Senior Fitness Test battery developed and revised by Rikli and Jones (79). The lower body strength is determined with the “*30 second’s chair-and-stand test”* (CST) that measures the total number of stands completed in 30 s. The upper-body strength is determined with the “*30 seconds Arm-curl test”* (ACT) that measures the total number of arm curls executed in the 30-s. The aerobic endurance is determined with the “*2-minute step test”* (2ST) that measures the number of full steps completed in 2 min, raising each knee to a point midway between the patella (kneecap) and iliac crest (top hip bone). Score is the number of times the right knee reaches the required height. To assess lower-body flexibility, the “*chair sit-and-reach test”* (CSR) measures the maximum reach as forward as possible toward or past the toes. The upper-body flexibility is determined with the “*back scratch test (BST)”* that measures the distance of overlap or between the tips of the middle fingers of the back. For the abovementioned each test, there is cut-off values adjusted for sex and age, which will be analyzed as continuous variables.

## Anthropometric Measures Assessment

The anthropometric measurements will be applied in four different moments following standardized procedures (80): in the beginning of the project for the data collection of the cross-sectional study and at three time-points during the intervention study. Measurements will take place in a separate room in order to give some privacy to the participants. Body mass will be determined using a portable scale (Seca^®^, model 770, Germany) with a precision of 0.1 kg. Waist circumference will be measured using a retractable glass fiber tape measure (Hoechstmass-Rollfix^®^, Germany) with a precision of 0.1 cm. Stature will be determined using a portable stadiometer (Seca Bodymeter^®^, model 208, Germany) with a precision of 0.1 cm.

## Characterization of the Exercise Programs

The development of the exercises programs will include the selection of intervention programs, defining the types of exercise, conducting literature review, consultation with specialists from each exercise program, presentation of the final version of the chair-based exercise program design, and beginning of implementation of the pilot study in care centers. All classes, of each exercise program, will be administered by two instructors. The main guidelines of exercise prescription recommended by the American College of Sport Medicine Science (ACSM) for older adults (81) will be followed. In addition, recent guidelines for exercise prescription in groups, with the support of a chair will be followed (82). The chair-based group exercise classes can vary in intensity from vigorous chair aerobics designed to provide muscle conditioning and aerobic benefits for healthy adults to movement that concentrates or maintaining a basic level function for older participants (82, 83). Music will not be used during the sessions, since the objective is to test the influence of exercise without music for some cognition parameters and therapy of music alone (84) or combined with physical exercise (85) can positively influence cognition. All exercise programs will have the same number of sessions. Detailed exercise description is presented in Table 1.

**Table 1:** **Overview of the two phases of chair-based exercise programs intervention and some specifics exercises**.

Modality	Warm-up phase^a^ (7 min.)	% HR	Main part of the workout/conditioning phase (30 min.)	% HR	Cool-down phase (7 min.)	% HR
		RPE		RPE		RPE
**Phase I – the first 14 weeks**						
AEW	General body mobilization and dynamic stretching chair-based exercises	~50–60 1–3	Aerobic walking activity: divided in 7–10 sets. They will be held a series containing 4–8 chair-based aerobic exercises. After performing 2–3 sets, the participants will be encouraged to combine the walk around the gym-room during 2–3 min. Walking time may be increased gradually during the program. Example of exercises: (1) chair-based sit and reach, leg extension, and overhead reach and standing rear leg extension (2–3 sets × 10 reps); (2) 2-min walking; (3) arm rising, hip marching, chair stand, and upper-body twist; (4) 2-min walking	~60–70 3–4	General body mobilization and static stretching chair-based exercises	~50–55 1–2
EB-RT	Eight exercises performed in two sets of six repetitions (reps) for general body mobilization	~50–60 1–3	Muscle-strengthening activity: 8–10 exercises using the first level of elastic Thera-band. Will consisted in to pull-up the elastic-band for 10 reps × 2 sets, with the concentric phase for 1 s and eccentric phase for 2 s with 45 s rest between sets and exercises. Examples of exercises: (1) front squat, (2) unilateral hip flexion in the chair, (3) bench over row, (4) chest press, (5) standing reverse fly, (6) spine twist extension arm, (7) shoulder press/twist arm front position, (8) frontal total raiser, (9) biceps arm curl stand/chair, and (10) overhead triceps exertion	~60–70 3–4	General body mobilization and static stretching chair-based exercises	~50–55 1–2
YTF	Standing or sitting exercises of joint mobilization and exercises to promote respiratory body awareness	~40–50 1–2	Standing or sitting practice of *aˉsanas* and postures sequences. Ex: (1) “Seated Forward Bend” (*Paschimottanasana*), (2)“Butterfly” (*Baddha Konasana*), (3) “Seated Spinal Twist” (*Ardha Matsyendrasana*), (4) “Cow face pose” (*Gomukhasana*), (5) “Cat” (*Cakravakasana*), (6) “Child’s Pose” (*Balasana*), (7) “Snake” *(Bhujangasana*), (8) “Child’s Pose with arms extended” (*Utthita Balasana*), (9) “Side Bending Stretch” (*Tiryaka Tadasana*), (10) “Dorsal Torsion” (*Kati Chakrasana*), (11) “Triangle” (*Trikonasana*), and (12) “Eagle” or “Half eagle” (*Garudasana*)	~50–60 2–3	Sitting or lying respiratory body awareness exercises, located massages, exercises for muscle relief, and meditation and vocalization	~40–45 1–2
**Phase II – the last 14 weeks**						
AEW	General body mobilization, dynamic flexibility chair-based exercises, and 3 min of “easy walking”^b^	50–60 1–3	The progress of the program will be followed according to aerobic endurance activity guidelines, therefore basing on the inclusion of more difficult and complex chair-based aerobic exercises and challenges sequences. Additionally, it will be increased walking time and placed obstacles during the walking route (cones, floor markers, arcs) to work handedness, changes in direction and coordination	60–70 3–4	1–2 min easy walking, general body mobilization, and static stretching stand exercises	50–55 1–2
EB-RT	Six exercises (3 sets × 6 reps) for general body mobilization and dynamic stretching	50–60 1–3	The progress of the program will be grounded on the muscle-strengthening activity guidelines, therefore basing on the inclusion of more exercises sets. Also, the same exercises in the Phase I will be used, but with more complex progressions. Example: 2–3 sets × 10 reps of front Squat in the chair + frontal raiser	60–70 3–4	General body mobilization and static stretching chair-based exercises	50–55 1–2
YTF	Standing or sitting exercises of joint mobilization and exercises to promote respiratory body awareness	~40–50 1–2	The progress of the program will be grounded on the Hatha Yoga philosophy, therefore basing on the inclusion of more difficult and complex aˉsanas and challenging sequences. Postures will be modified or added, sequences should have more postures with more difficult transitions, and the goal range of each posture will be increased	~50–60 2–3	Sitting or lying respiratory body awareness exercises, located massages, exercises for muscle relief, and meditation and vocalization	~40–45 1–2

*CBE, chair-based exercises; RPE, rated perceived exertion; % HR, percentage of the estimated maximal heart rate; Reps, number of repetitions; Rest, interval recovery*.

*^a^Specific exercises for different body segments, i.e., neck and shoulders, back and chest, waist and legs*.

*^b^Easy walking, 2–3 min of walking around the fitness room*.

## Chair Elastic-Band Strength Exercises

The chair elastic-band strength exercises (CSE) consist of an exercise class performing a determined number of sets, repetitions, cadence of execution, and rest between sets using a Thera-band^®^ elastic bands exercise system (86), that takes into account the ACSM guidelines for muscle-strength exercises prescriptions for older populations (81). The session consist of six exercises for body mobilization and dynamic stretching (5 min warm-up); between 8 and 10 elastic-band exercises using the three first levels of elastic bands (yellow, red, and green), for the development of muscle-strengthening activity (20–30 min); five easy stretching exercises to promote cool down lasting 15 min. We will expect real effort to be 60–85% of maximum heart-rate values recommended by the ACSM as the work intensity for the older people submitted to exercise programs. This would allow the training stimulus dosage to be precisely controlled in both the session in progress and between different sessions (86).

## Chair Aerobic Exercises to Improve Walking

The classes of chair aerobic to improve walking exercises (CAW) include activities that involve minutes of walking, chair-based sit, and reach exercises and activities for upper and lower body members. The walking time is expected to increase gradually during the program (81). The session has a maximum duration of 45 min divided into three parts with the following characteristics: warm-up (5–10 min) six exercises for body mobilization and dynamic stretching, 7–10 specific exercises to improve walking in a sitting and standing position, lasting 20–30 min, and cool-down easy stretching exercises to promote cool down lasting 15 min. Exercise intensity will be measured with heart rate (HR) monitors and real effort is expected to be 60–85% of maximum HR.

## Chair Yoga Type Flexibility Exercises

The introduction of āsanas shall be made through a sequence of movements combined with breathing (87). This method allows the modification of postures itself and of how to “enter” and “leave” the postures, which simplifies working with limiting conditions in group classes with various levels of physical ability and can be reviewed according to the participants evolution (88). Additionally, the methods of design of the exercise programs include the ACSM guidelines for stretching exercises prescriptions for older populations (81). The global exercise intensity will be measured with HR monitors, and it is expected that the real effort will be 50–75% of maximum HR, the values recommended by the ACSM.

## Exercise Intensity Control

Heart rate monitors will randomly be used in five participants, during the exercise sessions in all exercise programs, as aid adjustment and control of training loads. For safety reasons, exercise intensity is indirectly predicted using the Karvonen’s formula to predict target HR but with maximal heart rate (HR_max_) being calculated using Franklin, Whaley, and Howley formula’s for older people (HR_max_ = 207 beat per minute − 0.7 × chronological age) (89).

## Exercise Adherence to the Intervention Protocol

The instructors will document attendance to each class and the levels of exercise adherence will be calculated using the number of sessions attended as a covariable in secondary analysis of the exercise results. Intervention groups are asked to attend at least 70% of the classes. The instructors and research team staff will be asked to motivate the participants every time an absence to two consecutive classes occurs (47).

## Data Analysis

The assumption of normality will be checked by the Kolmogorov–Smirnov test with Lilliefors’ (K–S) significance correction and by visual inspection of normality plots. In study 2, comparison between groups will be accomplished using Univariate Statistics, in particular the independent *T*-test for 2 paired samples, and one-way analysis of variance (ANOVA) for *K*-independent samples. To elucidate which pathways (as biological, psychological, and behavioral parameters) will play a significant role in the effects of exercise on cognitive function in the participants, we will conduct mediation analysis. In this case, hormonal mediation will be analyzed by including hormone levels as covariates in the models with cognitive and immunological outcomes, to see if they partially or fully explain the exercise effects (make exercise effects less or non-significant), where there are exercise effects. These analyses will be conducted using SPSS and R (www.r-project.org) at a significance threshold of alpha = 0.05, adjusted for multiple comparisons where relevant. Data analysis will include repeated measures ANOVA models for block designs of different exercise conditions (SPPS and R), and mixed effects models to include graded dose-dependent exercise effects whilst also allowing for repeated measures data (using R). ANOVA models require principally that variance between cells does not differ too strongly (not more than factor 2), which will be checked in the assumptions checking stage. Mixed effects models allow very flexibly for non-normal outcome data distributions, for example, binomial for accuracy data (e.g., correct/wrong answers on cognitive tests) or Poisson for count data. Biological and psychological data are also often log-normally distributed (e.g., hormone levels, reaction times), variables where this is applicable will be log-transformed if the resulting distribution shows a better match with a normal distribution. Model residuals will also be checked for deviations from normality. Because a large number of outcome variables will be collected in this study, variable grouping and compound score selection will be based initially on theoretical domains (90, 91). The between-subject SD for each dependent variable was used to convert the changes in all variables into standardized [Cohen effect size (ES)] changes in the mean. Using Hopkins as guide (92), ESs were considered as trivial (*d* ≤ 0.2), small (0.2 < *d* < 0.6), moderate (0.6 < *d* < 1.2), large (1.2 < *d* < 2.0), very large (2.0 < *d* < 4.0), and nearly perfect (*d* > 4.0). This will be confirmed by correlation matrices (all variables in a group should correlate with at least 50%) and principal component analyses where the first component should carry at least 70% of the variance.

## Discussion

This study will allow us to investigate which hormonal parameters are able to mediate the effects of exercise on immunity, psychological stress, and cognitive improvement and dementia risk in older people who are regularly active and which type of exercise is more effective in promoting immune and psychological health and cognitive improvement. The final goal is to develop exercise protocols that may lead to prevention of disease and a better quality of life. This study also sustains the hypothetical premise that exercise improves neurocognitive functions, following a current trend study in the field of aging (23, 59, 93). However, to the best of our knowledge, no large intervention study has yet been conducted on the effect of these exercise interventions on cognitive decline in subjects with MCI. The pilot study for this type of population is essential as it will reveal to what extent adherence to exercise suffers influence of psychosocial factors in chronic diseases (94, 95). It is important to monitor the effectiveness of the program, as evidence shows that a low cognitive profile may compromise the trainability of the participants (96). This study also shows a strong multidisciplinary approach, since it is prudent to investigate the combined effects of some independent variables. In addition, we will also look at the hypothetical premise that some objective measures have strong associations with subjective perception measures.

## Author Contributions

AT and JF designed and coordinated the research project. GF and MC drafted the paper. All the authors have made a substantial contribution in their respective areas of expertise, critically revised the work, approved the final version, and agreed to be accountable for all aspects of the work.

## Conflict of Interest Statement

The authors declare that the research was conducted in the absence of any commercial or financial relationships that could be construed as a potential conflict of interest.

## Abbreviations

α-amy, salivary alpha amylase; 2ST, 2-minute step test; 8-UGT, 8-foot up-and-go test; ACSM, American College of Sport Medicine Science; ACT, 30 seconds arm-curl test; AD, Alzheimer disease; ADL, Katz index of independence in activities of daily living; BDNF, brain-derived neurotrophic factor; BST, back stretch test; CAW, chair aerobic to improve walking exercises; CCI, Charlson comorbidity index; CRP, C-reactive protein; CSE, chair elastic-band strength exercises; CSHS, Centers for Social and Health Care Support; CSR, chair sit-and-reach test; CST, 30 second chair-and-stand test; CYE, chair yoga type flexibility exercises; DHEA, dehydroepiandrosterone; DST, digital span test; ES, effect size; FES, falls efficacy scale; HADS, hospital anxiety and depression scale; HPA axis, hypothalamic–pituitary–adrenal axis; HR_max_, maximal heart rate; HVLT, Hopkins verbal learning task; IADL, Lawton independence activities day living scale; IFN-γ, interferon gamma; IL-1β, interleukin-1 beta; IL-10, interleukin 10; IL-6, interleukin-6; K–S, Kolmogorov–Smirnov test with Lilliefors; MCI, mild cognitive impairment; MMSE, mini mental state examination; MOCI, moderate cognitive impairment; NCP, normal cognitive profile; PA, physical activity; PF, physical fitness; PRO-HMECSI, hormonal mediation of exercise on cognition, stress, and immunity project; PSS, perceived stress scale; RSES, Rosenberg self-esteem scale; SCI, severe cognitive impairment; sCoR, salivary cortisol; sIgA, salivary immunoglobulin-A; SMT, symbol modality test; SWLS, satisfaction with life scale; TNF-α, tumor necrosis factor alpha; VFT, verbal fluency.
